# Practical Notes on Diagnosis and Treatment

**Published:** 1910-10-29

**Authors:** 


### Practical Notes on Diagnosis and Treatment.

**Transient Heart Murmurs.**

Transient aortic systolic murmurs are not at all uncommon in healthy but nervous individuals, especially candidates for life insurance. In such a case, if the candidate sit down quietly, and his attention be drawn to other things, in a short time the murmur will be found to have disappeared. The condition is probably due to a temporary dilatation of the aorta, in consequence of the excitement; this has the effect of making the aortic orifice relatively narrowed.—*Dr. Samuel West.*

**Perforation.**

In perforated gastric ulcer, after the first shock has passed off, before the fever of peritonitis supervenes, there may be a deceptive lull, in which the depressed temperature returns to normal and the feeble pulse resumes its steady beat. If the pulse and temperature, especially the latter, fail as criteria of the seriousness of the case, a most important point to remember is that in perforation, during the stage of reaction, when other signs are sometimes absent, muscular rigidity of the abdomen can nearly always be detected.—*Mr. S. Handley.*

**Coccygodynia.**

Before making this diagnosis always examine the anus carefully as well as the coccyx, and satisfy yourself that there is not a painful pile or an anal ulcer.—*Mr. Bland Sutton.*

